# Structure-based drug design of a new chemical class of small molecules active against influenza A nucleoprotein in vitro and in vivo

**DOI:** 10.1371/currents.RRN1253

**Published:** 2011-08-07

**Authors:** Peter Fedichev, Roman Timakhov, Tim Pyrkov, Evgeny Getmantsev, Andrey Vinnik

**Affiliations:** ^*^Chief Scientific Officer, Quantum Pharmaceuticals; ^†^Pharmacology, Biotechnology, Molecular biology, Molecular oncology; ^‡^Researcher, Quantum Pharmaceuticals Moscow; ^§^Quantum Pharmaceuticals, Researcher, Software Developer and ^¶^CEO, Quantum Pharmaceticals Moscow

## Abstract

We report preliminary results and a summary of a bottom-up approach to identify new, active, nontoxic, small-molecule antivirals designed to have а novel mechanism of action. We employed the procedure to identify 3-mercapto-1,2,4-triazoles derivatives as potential NP inhibitors in silico and subsequently demonstrated the in vitro efficacy of the molecules against various strains of the influenza A virus. The most efficacious compounds were successfully tested in an in vivo influenza challenge experiment.

## Introduction 

The drugs used against influenza can be classified into two broad groups depending on whether the compound acts on a host factor of the human cell or on virus target proteins (e.g., see [1] for a review). The host factor drugs are less prone to the development of drug resistance and usually have a wide range of antiviral activities [2] [3] [4] [5]. Although this approach is quite popular, attacking human cell targets may influence essential functions of the organism and may therefore require sophisticated studies regarding the mechanism of action (MoA) and adverse effects. 

 Commonly used virus-targeting compounds such as amantadine and rimantadine [6] inhibit the ion channel M2 [7] [8] and disrupt virus entry into target cells. Modern treatments such as zanamivir and oseltamivir (Tamiflu) inhibit the action of the membrane protein neuraminidase [9]. Unfortunately, all of these treatments lead to the emergence of drug resistance and side effects [10] [11] [12] [13] [14].

 The development of novel antiviral medications is complicated by an extreme degree of genetic variability of the pathogen and its ability to cross interspecies barriers [15] [16]. A plausible solution involves the development of a targeted antiviral compound that is highly active against a specific virus target. The selected target should concomitantly be highly conserved within the virus population with little similarity to any human protein. 

 An example of the approach involves the selective use of nucleoprotein (NP), which is one of the most conserved proteins within the influenza virus genome and has recently been suggested to be a promising drug design target [1] [17]. We applied a sequence of in silico screening tools [18] using virtual libraries of small molecules and identified the derivatives of 3-mercapto-1,2,4-triazoles as potential NP inhibitors. The top predicted binders were confirmed to be effective against various strains of influenza A in vitro. The most active compound demonstrated sufficient efficacy in terms of animal protection in the influenza challenge model in mice. 

##  Results 

 The 3D structure of the monomeric NP was taken from a previous study [19] and corresponds to an H5N1 virus (PDB code 2Q06). Two binding cavities of sufficient volume (exceeding 400A^3) for the subsequent docking were found using the flood-fill algorithm of PocketPicker [20]. The first site (referred to as Site 1) is located near the epitope sequence I265-S274 from [21]. The other site, Site 2, is situated next to the epitope sequence R174-K184 from a previous study [21].

**Figure:**
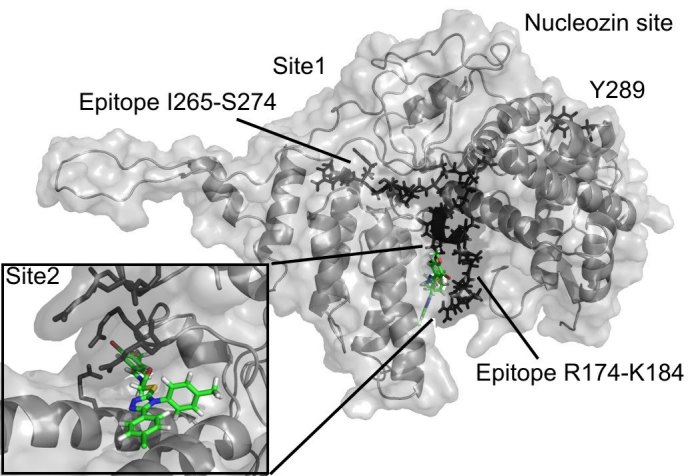


Figure 1. The compound F66 docked to influenza NP (see Results and Discussion sections for the details). 

NP inhibitors were identified via molecular docking of a specially prepared small molecules library (see Materials and Methods section) to Sites 1 and 2. To confirm the computed activity of the top ten predicted binders, we measured the cytopathic effect (CPE) of the influenza A/Wisconsin/67/2005(H3N2) virus in the presence of the studied compounds at a concentration of 5 μM in a plaque formation assay. Oseltamivir phosphate was used as a positive control. At least four different 3-mercapto-1,2,4-triazoles derivatives, all docked to Site 2 (see Figure 1), were determined to be active (see Table 1 for the summary of the results) in vitro.

**Table:** 

ID	Structure	# of Plaques /control	ID	Structure	# of Plaques /control
F66	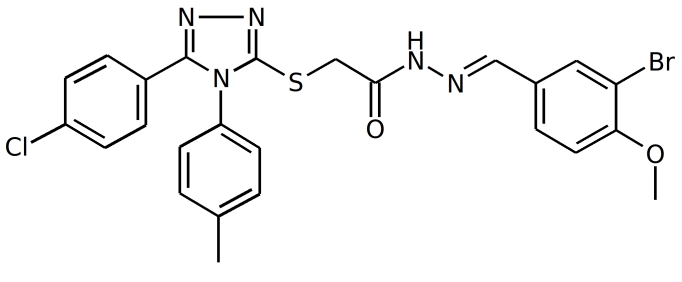	**0%, ** (0%,0%)	F66B	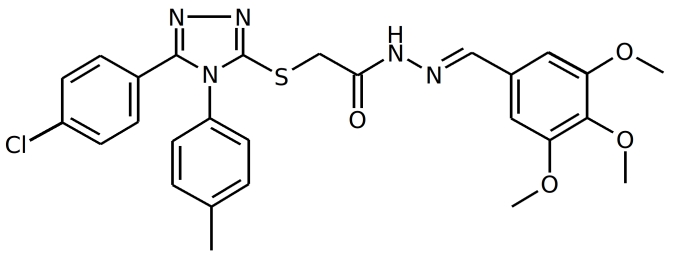	**4%**, (3%,6%)
F66A	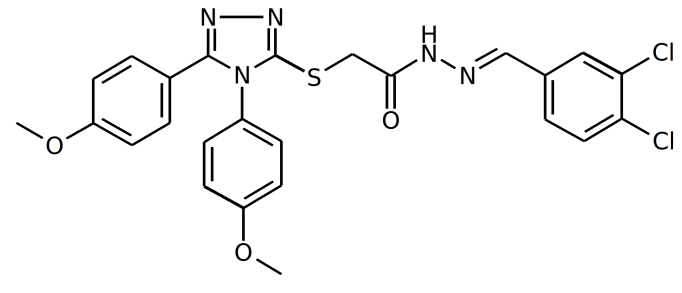	**5%**, (5%,4%)	F66C	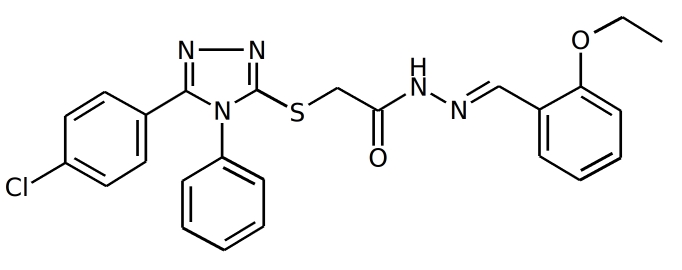	**4%**, (3%,5%)

Table 1. Plaque forming assay of the top 3-mercapto-1,2,4-triazoles derivatives against  A/Wisconsin/67/2005(H3N2). The numbers in brackets represent the results of the two subsequent single concentration tests.

The most active compound in the series, (3-bromo-4-methoxyphenyl)methylidenehydrazide of 5-(4-chlorophenyl)-4-(methylphenyl)- 1,2,4-triazole-3-ylthioglycolic acid, or F66, demonstrated concentration-dependent inhibition of the plaque formation, resulting in a cell protection effect estimate of EC50≲1 μM (see Figure 2).  

**Figure:**
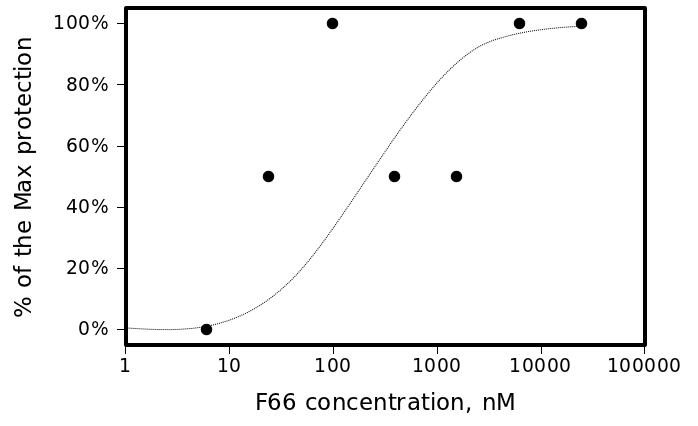
Figure 2. CPE of A/Wisconsin/67/2005(H3N2) virus in the presence of different concentrations of F66. The smooth line represents Michaelis-Menten estimate with Michaelis constant (EC50) of 250nM. 

The cytotoxicity measurement in HELA cell culture demonstrated no signs of cytotoxicity at concentrations up to 50 μM. In fact, we were not able to reach higher concentrations because of solubility issues, which indicated that the selectivity index (SI) well exceeded 50. 

 In another experiment against A/Aichi/2/68(H3N2), F66 also exhibited activity in the micromolar range. The hemagglutination activity (HA) inhibition efficacy of F66 exceeded the efficacy of rimantadine, which was used as a positive control in the same experiment (see Table 2). 

**Table:** 

Compound	Dosage	HA inhibition
F66	15 μM	75%
F66	10 μM	50%
F66	5 μM	50%
Rimantadine	50 mg/l	75%

Table 2. Efficacy of the F66 compound in-vitro against A/Aichi/2/68 (H3N2).

The cell-protection effect of F66 has been demonstrated in single-concentration measurements against a virus panel comprised of the virus strains targeted by the influenza vaccine for the season 2010-2011: B/Brisbane60/2008, A/NewCaledonia/20/99(H1N1), A/California/07/2009(H1N1) and A/Perth/16/09(H2N2). The results of the experiments are summarized in Table 3. F66 demonstrated a fair degree of cell protection. The EC50 can be estimated at approximately 1 μM against A/California/07/2009 (H1N1), approximately 5 μM against A/New Caledonia/20/99 (H1N1), and slightly higher than 5 μM against A/Perth/16/09(H3N2). There was no effect against the B/Brisbane60/2008 virus. All of the strains used in the experiment were sensitive to oseltamivir. A/California/07/2009 is a rimantadine-resistant strain.

**Table:** 

Virus	B/Brisbane60/2008	A/California/07/2009 (H1N1)	A/New Caledonia/20/99 (H1N1)	A/Perth/16/09 (H3N2)
Drug	Numbers of plagues in duplicate wells			
F66, 5 μM	**91%** (84%;98%)	**14%** (16%;13%)	**33%** (40%;25%)	**71%** (72%;70%)
Oseltamivir	**0%** (0%;0%)	**0%** (0%;0%)	**0%** (0%;0%)	**0%** (0%;0%)
No treatment	100%	100%	100%	100%

Table 3: Plaque formation by various influenza virus strains measured in the presence of F66 (5 μM) relative to an untreated control. The pair of values in brackets represents the results of the two successive measurements.

In a separate study, we measured the efficacy of the compounds from the F66 series at 5 μM against A/PuertoRico/8/34(H1N1). This strain is remantadine resistant, and thus, we were required to use a compound with a different MoA, such as ribavirin, as a positive control. We used the immunostaining of fixed cells  assay to detect the virus NP and evaluate the efficacy of the compounds. All four hits in the series, including F66 itself, were found to be more active than ribavirin under the same experimental conditions and at the same concentration.  

 In a similar experiment, we tested the efficacy of F66 against the A/Chicken/Kurgan/2005(H5N1) virus in a hemagglutination assay. The virus protein activity inhibition reached 50% at an F66 concentration range of 1 to 5 μM. A/Chicken/Kurgan/2005(H5N1) is the most virulent influenza strain that was obtainable for the experiments. 

 Given the promising in vitro results and apparent lack of toxicity, we tested F66 in influenza challenge experiments in mice. The animals were infected with the A/Aichi/02/68 (H3N2) virus corresponding to the 1-3 LD100 dosage (10-30 LD50). The substance was administered peroral (p.o.) either a few hours after the infection and subsequently once a day for the duration of the experiment (the emergency prophylaxis protocol) or once a day beginning 24 hours after the infection (the treatment protocol). We tested the two doses, 7 mg/kg and 22.5 mg/kg, of F66 daily. Two more well-known antiviral drugs, rimantadine and oseltamivir, were also used as positive controls. In parallel, we evaluated one animal group treated with a placebo (no drug) as a negative control and one uninfected group treated with F66 at 150 mg/kg daily as the toxicity control. The results of the study are presented below in Table 4. 

**Table:** 

Protocol	Treatment protocol		Emergency prophylaxis protocol	
Drug	Surviving animals, %	\begin{equation*}\Delta,\end{equation*} days	Surviving animals, %	\begin{equation*}\Delta,\end{equation*} days
F66, 7 mg/kg	21.1%	1.1	40%	2.7
F66, 22 mg/kg	31.6%	1.8	40%	2.7
Tamiflu, 25 mg/kg	94.7%	6.5	100%	5.8
Rimantadine, 30 mg/kg	45%	2.9	60%	3.9

Table 4. Efficacy of F66 against A/Aichi/02/68(H3N2) challenge in-vivo.The values Δ indicate the average life expectancy increase in the group relative to the infected control.  

The treatment protocol study of F66 demonstrated the animal protection effect of F66 in a dose-dependent manner. The maximum level of protection of 31.6% was achieved when the animals were receiving 22.5 mg/kg of the substance daily. The emergency prophylaxis protocol study demonstrated that F66 has similar and moderate protective efficacies at each of the doses of 7.5 and 22.5 mg/kg. The animals in the toxicity control group tolerated the compound well.

## Discussion

Both docking cavities identified by the floodfill algorithm are in close vicinity to and in close contact with the earlier suggested NP epitope sequences studied in a previous study [21] (see Figure 1). Site 2 occupies an arginine-rich region of the RNA-binding groove and forms extensive contacts with another NP-conserved epitope sequence R174-K184, which makes the site very attractive for targeting NP. Meanwhile, Site 1 has only minor contact with another epitope sequence, I265-S274. Surprisingly, both Site 1 and Site 2 are distant from the proposed binding site of the recent NP inhibitor nucleozin, which is marked by the residue Y289 [17] on Figure 1. 

 The demonstrated level of animal protection in treatments with F66 reached 40%, which is comparable to the protection provided by treatment with rimantadine in a similar experiment (60%). In the emergency prophylaxis study, the compound application increased the lifetime of the mice by 2.7 days. The results are inferior to the protection level achieved with Tamiflu, which is not surprising given that Tamiflu exhibited an efficacy that was more than two orders of magnitude higher in vitro. 

 The activity of F66 was first predicted in silico as a result of a docking model against influenza NP. Currently, we have limited knowledge regarding the MoA of the efficacy of the compound from the measurements in the compound series against the two different amantadine-resistant strains, A/Puerto Rico/8/34(H1N1) and A/California/07/2009(H1N1). In both of these cases, the activity of F66 was even better than that observed in the measurements against other influenza strains. This lets us conclude that  the F66 MoA most likely does not involve an interaction with the target of amantadine, M2. 

 F66 was effective against a number of influenza A strains but was considerably less effective against influenza B. This result could be explained if the target of the compound is actually NP. The active site that we selected for the docking is only 75% conserved between the influenza A and B viruses, and the difference in the experimental efficiency may thus be compatible with the suspected MoA of the compounds. 

 Among the influenza A strains, the least in vitro activity was demonstrated against H3N2 variations of the virus. Moreover, among the H3N2 strains we utilized in vitro, the efficacy difference between A/Wisconsin/67/2005 and A/Perth/16/09 was the largest. Unfortunately, the NP of A/Perth/16/09 has not yet been sequenced, and our activity data thus cannot be used to elucidate the MoA. To confirm NP as the target of the compounds, we need to perform a mutant escape assay and/or a direct binding assay using surface plasmon resonance (SPR) with a recombinant version of the protein. 

 The calculations show that F66 binds to Site 2, which is responsible for RNA binding. The binding site is fairly conserved, and, if proven, our approach may provide an alternate strategy for influenza NP inhibition. Despite its clear medicinal chemistry deficiencies, the compound was well tolerated and active in vivo. Although results reported here are very preliminary and a lot more further steps in lead optimization should be performed, we believe that the derivatives of F66 may eventually become effective oral agents for the treatment of influenza virus infections. 

## Materials and methods 

### Virtual compounds library design

 The molecules for the virtual screening were taken from the libraries provided by Alinda, Asinex, Chemdiv, Enamine and IBS. Together, they contain more than 3 million readily synthesized molecules. To reduce the calculations volume, the compounds library was clustered according to a previous study [22]. The measure of dissimilarity (the “distance”) between the molecules was determined by Tanimoto and similarly calculated with Daylight fingerprints [23]. The clustering parameters were chosen so that a typical cluster was comprised mostly of chemically similar compounds. We chose one representative molecule from each of the ~73,000 clusters with ten or more molecules. The inclusion of smaller clusters does not lead to more coverage of the libraries by the clustered molecules, and at the same time, it dramatically increases the total number of clusters. As a result, our cluster centroids library covered approximately 53% of the total number of molecules.

### Virtual screening

Computational techniques generally involve a balance between speed and accuracy [24]. To overcome the bottle-neck effect posed by the computationally demanding molecular dynamics (MD)-based methods, we used a previously described combination [18] [25] of the two approaches: a fast molecular docking for the generation of binding poses using a simplified potential of mean force (PMF, see e.g. [26]) followed by the molecular dynamics (MD) simulations to rank the ligands according to their binding affinities calculated using the linear interaction energies (LIE) approximation [27]. The MD simulations were performed in an AMBER/GAFF [28] [29] force field combined with a continuous model of the aqueous environment [30] [31]. The compounds with the top predicted binding affinities of greater than 10 μM were selected for subsequent in vitro tests.

### Influenza virus strains

The experiments with the virus strains A/Wisconsin/67/2005(H3N2), A/New Caledonia/20/1999(H1N1), A/Perth/16/2009(H3N2), A/California/07/2009 and B/Brisbane/60/2008) were performed at Virapur, US. The virus samples were taken from the Virapur collection. The strain A/Puerto Rico/8/34 (H1N1) was received from the collection of the Research Institute of influenza, Russian Academy of Medical Sciences, St. Petersburg, Russia. The virus strain A/Aichi/2/68 (H3N2) was taken from a collection of Branch of FGU "48 The Central Scientific Research Institute of the Russian Defense Ministry", Russia. The virus strain A/Chicken/Kurgan/2005 (H5N1) was received from the Institute of Poliomyelitis and Viral Encephalitis, Russia.

### Plaque assay

Incubation, infection and staining: In total, 96-well dishes were seeded 20 hours previously with a known concentration of MDCK cells. A 100µl volume of a drug dilution was carefully placed on each monolayer in quadruplicate and incubated on the monolayers for one hour. All of the virus stocks had been previously aliquoted and tested for the number of plaque-forming units per volume. Drug solutions were incubated on the cells for one hour, andapproximately 50-75 plaque-forming units of each influenza virus were subsequently added to each well. The virus was allowed to adsorb to the monolayer for a defined time. Following the incubation of the virus, the virus inoculum was suctioned off of the monolayer, and the appropriate dilute drug solution, to which 0.4% agarose had been added, was carefully overlaid on the appropriate monolayer. 

 Cultures infected with viruses A/Wisconsin/67/2005, A/New Caledonia/20/1999 and A/Perth/16/2009 were incubated at 34 °C for 48 hours and monitored for plaque size. The monolayers were observed under the inverted microscope and graded for the presence of the influenza cytopathic effect (CPE). Metabolically active cells were stained with 3-[4,5- dimethylthiazol-2-yl]-2,5-diphenyltetrazolium bromide (MTT). 

 Cultures infected with the viruses B/Brisbane/60/2008 and A/California/07/2009 were incubated at 34°C for 68 hours and monitored for plaque size. Cultures were stained to easily visualize plaques after 68-72 hours of virus incubation. Metabolically active cells were stained with MTT. 

 Counting plaques: Plaques that consist of infected cells originating from one infectious virus are very metabolically active in the second and third day of infection. Stained plaques were counted manually, and the number of plaques observed in the duplicates of each drug was recorded. In some instances, plaque size was reduced with drug treatment, but the number of plaques was not reduced.

#### Virus proteins detection by immunostaining of fixed cells.

MDCK cells (ATCC CCL 34) were used. The compounds under investigation were dissolved in minimum essential medium (MEM) to a concentration of 5 µM. The solutions were applied to MDCK cells and incubated for 1 hour at 37 °C followed by infection with the influenza virus in two doses (1 and 10 EID50/0.1 ml) in the medium. Control wells did not contain the compounds. Ribavirin was used as a positive control. Viruses were cultivated at 37 °C for 24 hours followed by an ELISA assay. 

For this purpose, cells were washed three times with phosphate-buffered saline (PBS) of pH 7.6, fixed in 80% cold acetone for 10 min at -10 °C, washed for 5 min with distilled water and air dried. Monoclonal antibodies against influenza virus NP protein were diluted to 5 µg/ml in 5% fat-free milk in PBS and incubated with fixed cells for 1 hour at 37 °C. After washing three times with PBS, cells were incubated with diluted 1:10,000 goat anti-mouse horseradish peroxidase-conjugated antibodies (Sigma, St. Louis MO) for 1 hour at 37 °C. Unbound antibodies were removed by washing three times in PBS, and a color reaction was developed by adding 3,3',5,5'-tetramethylbenzidine and 0.03% H2O2 in a 0.1 M acetate buffer of pH 5.0. The reaction was stopped with 2N H2SO4, and the optical density of the wells was measured at a wavelength of 450 nm. The ELISA reaction was considered positive if the optical density (OD450) exceeded twice the value of the corresponding uninfected control cells. The compounds were considered to be active if OD450 was two or more times lower than that in the control.

### Hemagglutination assay

 MDCK cells were used. Dilutions of the compounds under investigation were prepared in MEM. The solutions were applied to MDCK cells followed by infection with influenza virus А/Aichi/02/68 (H3N2) at 0.1-0.01 TCID50 and Incubated for 24-72 h at 37 °C until the development of a cytopathic effect in the control. The level of hemagglutinin activity in the lysate of the infected cells was determined using standard procedures [32].

### Anti-influenza activity in animal models 

Albino mice weighing 10-12 g were obtained from the 48 MD Russia vivarium research center. The drug was administered in two doses, 7 and 22.5 mg/kg bw. Each group of mice included 20 animals. The virus A/Aichi/2/68 (H3N2) was administered intranasally at 10-30 50% lethal influenza virus doses (LD50) under slight ether anesthesia. The following treatment scheme was used: 0.2 mL of a drug water solution was administered p.o. every 24 hours after infection for 6 days. The following emergency prophylaxis scheme was used: a 0.2 mL drug water solution was administered i.g. 1-2 hours after infection, and every 24 hours afterwards for 6 days. The animals were observed for 14 days, and deaths in the control and experimental groups were reported every day. Based on these data, the degree of animal protection was calculated.

## Acknowledgements

The authors wish to thank Dr A. Kadushkin (Quantum Pharmaceuticals) for assistance in resolving numerous chemical issues, Dr. S. Borisevich (FGU "Virology Center", Institute 48 MD, Russia) for the in vivo work and the Virapur (US) team for the in-vitro efficacy studies. 

##  Funding information 

The work is funded by Quantum Pharmaceuticals (Russian Federation).

## Competing interests 

The authors are employed by Quantum Pharmaceuticals. The Russian Patent Office application covering the reported activity of 3-mercapto-1,2,4-triazoles derivatives against influenza has been filed.  
